# Trends in socioeconomic inequality of periodontal health status among Dutch adults: a repeated cross-sectional analysis over two decades

**DOI:** 10.1186/s12903-021-01713-x

**Published:** 2021-07-15

**Authors:** An Li, Jan Hendrik Vermaire, Yuntao Chen, Luc W. M. van der Sluis, Renske Z. Thomas, Geerten-Has E. Tjakkes, Annemarie A. Schuller

**Affiliations:** 1 Department of Periodontology, Center for Dentistry and Oral Hygiene, University Medical Center Groningen (UMCG), University of Groningen, Groningen, The Netherlands; 2 Department of Child Health, The Netherlands Organization for Applied Scientific Research (TNO), Leiden, The Netherlands; 3 Medical Statistics and Decision Making, Department of Epidemiology, UMCG, University of Groningen, Groningen, The Netherlands; 4 Department of Dentistry, Radboud Institute for Health Sciences, Radboud University Medical Center, Radboud University Nijmegen, Nijmegen, The Netherlands

**Keywords:** Socioeconomic inequality, Trends, Oral hygiene, Periodontal health, The Netherlands

## Abstract

**Background:**

Studies exclusively focusing on trends in socioeconomic inequality of oral health status in industrialized countries are relatively sparse. This study aimed to assess possible differences in oral hygiene and periodontal status among people of different socioeconomic status (SES) in the Netherlands over two decades.

**Methods:**

A repeated cross-sectional analysis of 3083 participants aged 25–54 years was conducted on the Dutch National Oral Health Surveys of 1995, 2002, 2007, and 2013. Plaque-free was defined according to the Simplified Oral Hygiene Index (OHI-S = 0). Periodontal status was classified in two different ways, either periodontal health/disease (probing pocket depth index [PDI] = 0/ ≥ 1) or with/without deep pockets (PDI = 2). We used the regression-based absolute and relative effect index to measure the absolute and relative socioeconomic inequalities. Multivariable logistic regressions were used to explore temporal trends in oral hygiene and periodontal status by low- and high-SES groups.

**Results:**

Age-standardized percentages of individuals with plaque-free increased in the whole population from 1995 to 2013 (12.7% [95% CI 10.5–14.9] to 28.1% [24.8–31.5]). Plaque-free showed significant socioeconomic differences in absolute and relative inequalities in 2007 and 2013. Between 1995 and 2013, age-standardized percentage of periodontal health increased (from 51.4% [48.1–54.7] to 60.6% [57.0–64.1]). The significant absolute inequalities for periodontal health were seen in 2002 and 2013. The relative scale presented a similar pattern. Regarding deep pockets, there was little difference in the age-standardized overall prevalence in 1995 versus 2013 (from 6.5% [4.9–8.2] to 5.4% [3.7–7.0]). The significant absolute and relative inequalities in deep pockets prevalence were found in 1995. Yet, all interaction terms between survey year and SES did not reach significance (plaque-free: *P* = .198; periodontal health: *P* = .490; deep pockets: *P* = .678).

**Conclusions:**

Socioeconomic inequalities in oral hygiene and periodontal status were present in the Netherlands in the last two decades.

## Background

Periodontal disease is one of the most prevalent oral diseases; given that the severe case affected 796 million people worldwide and is associated with a variety of systemic conditions, it is considered a public health problem worldwide [1, 2]. Routine oral hygiene and effective plaque control have been identified as critical tools for achieving good periodontal health [3]. In recent decades, the periodontal health of adults has improved to varying degrees in Switzerland, Sweden, and Norway [4–7]. In contrast, the proportion of the severe periodontitis group did not decrease. Despite some progress achieved in periodontal prevention and care, social inequality in terms of periodontal health persists, and those on the lower rungs of the socioeconomic ladder still carry a substantial burden of periodontal disease and experience the poor oral health-related quality of life [8].

Socioeconomic inequality is thought to be socially unfair. More importantly, reducing disease burden of the lower socioeconomic population will largely improve the average health status of the overall population [9]. Therefore. reducing these inequalities is one of the primary goals of public health policies [10]. An understanding of the effects of socioeconomic changes on oral health over time can influence policymakers when revising policies, allocating budgets, and attempting to improve health quality [11]. Trends in terms of socioeconomic inequalities in children’s oral health and adults’ self-reported oral health, restorative dental treatment need, and tooth loss have been reported across the globe [12–17]. However, few nationally representative data have explored the effect of socioeconomic differences on temporal trends in oral hygiene and periodontal status among adults over time.

The existence of a national database on oral health in the Netherlands provides a unique opportunity to study trends in oral health disparities related to inequalities in socioeconomic status (SES) [18]. Hence, we examined the 1995–2013 trends in oral hygiene and periodontal status among Dutch adults and attempted to describe the possible inequalities in oral health linked to SES during the last two decades.

## Methods

### Data source and study population

The data used in this study were retrieved from a database developed by the Netherlands Organization for Applied Scientific Research (Toegepast Natuurwetenschappelijk Onderzoek, TNO). The Dutch National Oral Health Survey series has continuously gathered information on the oral health and oral health behavior of non-institutionalized civilian adult residents since 1995 [18]. The survey series was designed as a repeated cross-sectional study of the oral health status of the Dutch population over time based on a variety of oral health measures. More specifically, the Dutch National Oral Health Survey series used in this study were conducted among people living in the city of ‘s-Hertogenbosch, the Netherlands. This population is considered to be representative of the general Dutch population in terms of demographic distribution [19]. Under the National Health Care Institute's authority, Health Insurance Fund (HIF) companies were asked to provide the names and addresses of their clients. Since the HIF system focused on the relatively lower-income population before 2006 [20], people with low SES could be overrepresented. Therefore, the analyses were performed in the whole population and in the sub-populations stratified by SES. The stratification method facilitates making comparisons and conducting trend analyses about oral hygiene and periodontal status over time, in line with the approach adopted in a previous study on caries experience [13]. Central Committee on Research Involving Human Subjects concluded that no ethical approval was required for data collection for this purpose. Furthermore, it was determined that all of the requirements of the Personal Data Protection Act had been met (No. m1501261).

**Fig. 1:**
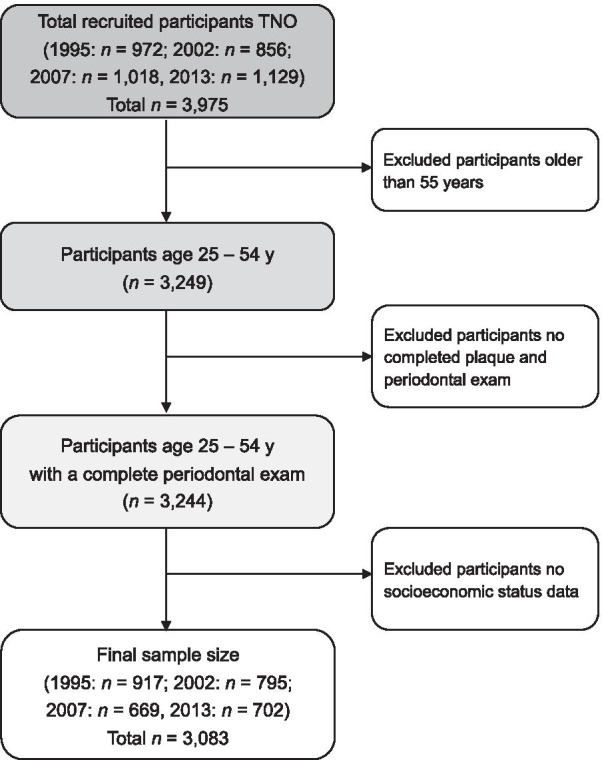
Flow chart indicating the subset of participants from 1995 to 2013 Dutch National Oral Health Survey included for analysis

Data from four TNO survey cycles conducted between 1995 and 2013 were used. In 1995 and 2002, the sample included only persons aged 25–54 years [21, 22]. In 2007 and 2013, the scope was widened to 74 years [23, 24]. To conduct trend analyses in equal groups, we only included data of participants aged 25–54 years in this study. Information on sociodemographic characteristics and clinical examinations were from four surveys (1995, 2002, 2007, and 2013) while excluding those who were lacking dental measurement or SES data. In total, 3083 respondents were included in the present study (see Fig. 1).

### Socioeconomic status and covariates

Educational attainment was used as a proxy for socioeconomic status [13]. The level of education was divided into “low” (only having completed primary school or having received a lower-level general secondary education) and “high” (having received a higher-level general secondary education or tertiary school) [25, 26]. Demographic variables, including age (25–34, 35–44, 45–54 years), gender and ethnic background, were collected via questionnaire and interview. Ethnicity (native/non-native Dutch) was determined on the participant’s birth country (the Netherlands versus any other country). Oral hygiene behaviors included the time since the last dental visit, frequency of daily teeth brushing, using dental floss, using tooth pick, and mouthwash. We considered these covariates as potential confounders that were controlled in the multivariable regression models.

### Assessment of oral hygiene and periodontal status

Oral health examinations were performed by a trained and qualified team of dentists and dental hygienists in a mobile research facility located in the municipalities in which the participants lived. The comprehensive oral health assessment included dentition status assessment, oral hygiene examination, and periodontal screening. Teeth missing (edentulous areas) or replaced by dental implants were recorded as lost teeth. To assess oral hygiene, the simplified oral hygiene index (OHI-S) was used to quantify dental plaque accumulation [27]. OHI-S was scored based on six index teeth: the buccal surfaces of teeth 16, 11, and 26 and the lingual surfaces of teeth 31, 36, and 46. Details concerning the criteria used in the OHI-S scoring can be found in Table 1. Oral hygiene was defined as “good” if all index teeth had OHI-S = 0, “fair” if at least one tooth with OHI-S = 1, and “poor” if at least one tooth with OHI-S = 2–3. In the current study, good oral hygiene (i.e., plaque-free [OHI-S = 0]) was considered as a binary outcome variable.

**Table 1:** Assessments and definitions of oral hygiene and periodontal status

Simplified Oral Hygiene Index (OHI-S)	Examination protocol	Buccal surfaces of teeth 16, 11, 26
		Lingual surfaces of teeth 31, 36, 46
	Score	0: no plaque
		1: plaque on the gingival third of the surface
		2: plaque on the middle third of the surface
		3: plaque on the occlusal third of the surface
	Definition	Plaque-free was defined as all index teeth had OHI-S = 0
Pocket Depth Index (PDI)	Examination protocol	Random half–mouth protocol
		Buccal surfaces of upper jaw
		Lingual surfaces of lower jaw
	Score	0: < 3.5 mm
		1: 3.5 – 5.5 mm
		2: > 5.5 mm
	Definition	(1) Periodontal health was defined as all assessed teeth had pocket depth < 3.5 mm (PDI = 0)
		(2) Deep pockets was defined as any assessed tooth had pocket depth > 5.5 mm (PDI = 2)

Periodontal status was assessed by probing pocket depth index (PDI), which is a standard periodontal parameter [18]. Periodontal examination was conducted on each tooth in two randomly selected quadrants (depending on the research ID number: ending with an odd number: we examined the 2nd and 4th quadrants; In case of an even number, we checked 1st and 3rd quadrants). This approach could minimize the burden of examination in large epidemiological studies. As third molars were excluded, a maximum of 14 teeth per individual could be examined. Three sites (buccal sites of maxillary vs. lingual sites of mandibular) were examined for each tooth using a Community Periodontal Index (CPI) probe recommended by the World Health Organization [28]. The highest score was recorded per tooth. The PDI scores were recorded as follows: PDI = 0, pocket depth < 3.5 mm; PDI = 1, pocket depth of 3.5–5.5 mm; and PDI = 2, pocket depth > 5.5 mm (Table 1). We defined those individuals with PDI = 0 (all teeth have probing pocket depth < 3.5 mm) as constituting the periodontal health group [29, 30]. Moreover, we defined PDI = 2 (≥ 1 tooth with probing pocket depth > 5.5 mm) as deep pockets group. These approaches allowed for binary outcome variables [29, 30].

### Statistical analysis

We calculated the means or the percentages of chosen population characteristics by survey year. The differences between survey years were analyzed by Chi-square test and one-way ANOVA. We calculated age-standardized percentages of oral hygiene (OHI-S = 0, 1, 2/3) and periodontal status (PDI = 0, 1, 2) using the direct method [31] with the data from Dutch population of 2006 as the standardized population. The age-standardized percentages with 95% confidence intervals (CIs) were presented for each study period.

We studied trends in socioeconomic inequality of oral hygiene and periodontal status in two steps. First, socioeconomic inequality was tested on the absolute and relative scales in each survey year (1995, 2002, 2007, 2013) [9]. We used multivariable logistic regressions to estimate the association of SES with oral health outcomes (i.e., plaque-free, periodontal health, and deep pockets). The logistic regressions were adjusted for potential confounders, including age, gender, ethnicity, SES, time since the last dental visit, number of teeth present, and oral hygiene behaviors (toothbrushing frequency, floss usage, and toothpick usage). Rate difference and odds ratio derived from the corresponding logistic model were used as the measure of absolute and relative effect [9, 32]. Rate difference was defined as the difference between the predicted probability of the oral health outcome in high-SES group and that in low-SES group. The predicted probabilities for each outcome were calculated using marginal-effect estimation [33]. 95% CIs for the rate difference were estimated using the delta method [34]. As a second step, the adjusted percentages (i.e., predicted probabilities) of oral health outcomes were presented graphically to evaluate temporal trends using pooling data from four waves. We performed interaction analyses for each oral health outcomes in which the survey year (as a continuous variable) and the interaction between survey year and SES were additionally added into the logistic model in step one. A statistically significant interaction term (survey year × SES) indicated an increase or disease in socioeconomic inequalities of oral health outcomes over time [35]. Complete case analysis was used for handling missing data. R Project for Statistical Computing (version 3.6.0) was used to perform all analyses.

## Results

### Characteristics of the study population

Participant characteristics aged 25–54 years by survey year are presented (Table 2). There were differences in the age and age composition of each survey year. The percentage of people with low-SES in 1995 and 2002 were higher than in 2007 and 2013. Compared to 1995 and 2007, the percentages of females in 2002 and 2013 were higher. Interdental hygiene behaviors (i.e., daily usage of floss and toothpick) improved over time. There were significant differences between survey years for these characteristics (*P* < 0.01).

**Table 2:** Characteristics of the Dutch population from the Dutch National Oral Health Survey 1995–2013

	1995	2002	2007	2013	*P* value^b^
	(*n* = 917)	(*n* = 795)	(*n* = 669)	(*n* = 702)	
Age year, mean (SD)	38.1 (8.1)	40.7 (8.0)	41.1 (8.5)	40.2 (8.6)	.000
Age group, *n* (%)					.000
25–34 y	353 (38.5)	211 (26.5)	177 (26.5)	217 (30.9)	
35–44 y	317 (34.6)	310 (39.0)	227 (33.9)	235 (33.5)	
45–54 y	247 (26.9)	274 (34.5)	265 (39.6)	250 (35.6)	
Gender, *n* (%)					.000
Male	450 (49.1)	318 (40.0)	304 (45.4)	284 (40.5)	
Female	467 (50.9)	477 (60.0)	365 (54.6)	418 (59.5)	
Ethnicity, *n* (%) ^a^					.000
Native	831 (91.1)	639 (80.8)	576 (86.5)	636 (90.6)	
Non-native	81 (8.9)	152 (19.2)	90 (13.5)	66 (9.4)	
Socioeconomic status, *n* (%)					.000
Low	579 (63.1)	601 (75.6)	309 (46.2)	264 (37.6)	
High	338 (36.9)	194 (24.4)	360 (53.8)	438 (62.4)	
Time since the last dental visit, *n* (%)^a^					.009
More than 1 year	57 (6.2)	71 (8.9)	73 (10.9)	63 (9.0)	
Less than 1 year	859 (93.8)	723 (91.1)	595 (89.1)	638 (91.0)	
Toothbrushing frequency, *n* (%)^a^					.000
< Once per day	254 (27.7)	218 (27.4)	122 (18.3)	182 (26.0)	
> Twice per day	663 (72.3)	577 (72.6)	545 (81.7)	517 (74.0)	
Floss usage, *n* (%) ^a^					.000
Non-daily	821 (89.5)	717 (90.2)	561 (84.4)	533 (75.9)	
Daily	96 (10.5)	78 (9.8v	104 (15.6)	169 (24.1)	
Tooth pick usage, *n* (%)^a^					.000
Non-daily	768 (83.8)	676 (85.0)	541 (81.5)	486 (69.2)	
Daily	149 (16.2)	119 (15.0)	123 (18.5)	216 (30.8)	
Number of teeth present, mean (SD)	24.1 (5.3)	24.0 (5.4)	25.3 (4.1)	26.4 (3.1)	.000

^a^Missing value for total study, ethnicity, *n* = 12 (0.4%); dental visit, *n* = 4 (0.1%); toothbrushing, *n* = 5 (0.2%); floss, *n* = 4 (0.1%); tooth pick, *n* = 5 (0.2%)

^b^All *P*-values were calculated with a two-sided significance level of .05

*SD* standard deviation

### Simplified oral hygiene index and pocket depth index from 1995 to 2013

The age-standardized percentages of the OHI-S and the PDI in the total population can be found in Table 3. Between 1995 and 2013, there was a decrease in poor oral hygiene (OHI-S ≥ 2: from 41.1% [95% CI 37.9–44.4] in 1995 to 10.9% [8.6–13.2] in 2013). Following this finding, the number of individuals with OHI-S = 0 showed an increasing trend over time. In contrast, no evident changes could be identified in the age-standardized percentages of individuals with deep pockets (PDI = 2) in the total population during the last two decades. Most of the changes in individuals with periodontal pockets (PDI = 1) occurred (from 42.0% [38.8–45.3] in 1995 to 34.0% [30.5–37.5] in 2013). This decreasing trend was accompanied by a steady increase (51.4% [48.1–54.7] to 60.6% [57.0–64.1]) in the proportion of individuals who exhibited periodontal health (PDI = 0, Table 3).

**Table 3:** Age-standardized percentage of oral hygiene and the periodontal status in the Dutch population from the Dutch National Oral Health Survey 1995–2013

Age-standardized percentage (95% CI)	1995 (*n* = 917)	2002 (*n* = 795)	2007 (*n* = 669)	2013 (*n* = 702)
Oral hygiene				
OHI-S = 0	12.7 (10.5–14.9)	8.9 (7.0–10.9)	20.6 (17.5–23.7)	28.1 (24.8–31.5)
OHI-S = 1	46.2 (42.9–49.4)	49.8 (46.4–53.3)	56.8 (53.0–60.6)	60.9 (57.3–64.5)
OHI-S = 2, 3	41.1 (37.9–44.4)	41.2 (37.8–44.6)	22.6 (19.4–25.8)	10.9 (8.6–13.2)
Periodontal status				
PDI = 0	51.4 (48.1–54.7)	46.5 (43.0–49.9)	56.3 (52.6–60.0)	60.6 (57.0–64.1)
PDI = 1	42.0 (38.8–45.3)	45.0 (41.6–48.5)	36.8 (33.1–40.5)	34.0 (30.5–37.5)
PDI = 2	6.5 (4.9–8.2)	8.5 (6.6–10.4)	6.9 (5.1–8.7)	5.4 (3.7–7.0)

Percentages of oral hygiene and the periodontal status were age-standardized by the direct method, with the age distribution of the Dutch population in 2006 as the reference

### Relative and absolute socioeconomic inequality of oral hygiene and periodontal status

Table 4 showed the relative and absolute socioeconomic inequality of plaque-free, periodontal health, and deep pockets in each survey year. For plaque-free, the significant absolute inequalities were observed in 2007 (absolute index = 8.4; 95% CI = 2.1, 14.7) and 2013 (absolute index = 8.6; 95% CI = 1.5, 15.7) while not in 1995 and 2002. When going from low-SES to high-SES, the adjusted percentage of plaque-free increased by 8.4 percentage points in 2007 and 8.6 percentage points in 2013, respectively. In the relative scale, the regression-based relative effect index presented a pattern similar to absolute inequality (1.698 [1.124–2.565] in 2007 and 1.561 [1.080–2.257] in 2013).

**Table 4:** Absolute and relative socioeconomic inequality in plaque-free, periodontal health, and deep pockets in adults aged 25–54 from the Dutch National Oral Health Survey 1995–2013

Estimate (95% confidence interval)	1995 (*n* = 917)	2002 (*n* = 795)	2007 (*n* = 669)	2013 (*n* = 702)
Plaque-free				
Regression-based absolute effect index ^a^	2.6 (− 1.7 to 6.9)	1.9 (− 2.1 to 6.0)	**8.4 (2.1 to 14.7)**	**8.6 (1.5 to 15.7)**
Regression-based relative effect index ^b^	1.308 (0.819 to 2.090)	1.292 (0.674 to 2.477)	**1.698 (1.124 to 2.565)**	**1.561 (1.080 to 2.257)**
Periodontal health				
Regression-based absolute effect index ^a^	3.7 (− 3.5 to 10.8)	**10.0 (1.6 to 18.4)**	4.0 (− 3.8 to 11.8)	**9.6 (1.8 to 17.4)**
Regression-based relative effect index ^b^	1.163 (0.873 to 1.549)	**1.516 (1.080 to 2.126)**	1.188 (0.852 to 1.656)	**1.516 (1.092 to 2.106)**
Deep pockets				
Regression-based absolute effect index ^a^	− **4.2 (**− **7.1 to** − **1.3)**	− 2.3 (− 5.9 to 1.3)	− 3.1 (− 5.4 to 0.7)	− 2.6 (− 5.6 to 0.3)
Regression-based relative effect index ^b^	**0.433 (0.216 to 0.869)**	0.730 (0.382 to 1.396)	0.618 (0.325 to 1.176)	0.585 (0.290 to 1.177)

All logistic regression models were adjusted for including age, gender, ethnicity, SES, time since the last dental visit, number of teeth present, and oral hygiene behaviors (toothbrushing frequency, floss usage, and toothpick usage). Low-SES was regarded as reference

^a^Regression-based absolute effect index was estimated according to the rate of oral health outcomes. Rate difference was defined as the difference between the predicted probability of the oral health outcome in high-SES group and that in low-SES group. Absolute inequality was considered significant if the 95% confidence interval did not cross zero. Bold indicates *P* value < 0.05

^b^Regression-based relative effect index was estimated according to the adjusted odds ratio of SES for oral health outcomes. Relative inequality was considered significant if the 95% confidence interval did not cross one. Bold indicates *P* value < .05

For the proportion of individuals having periodontal health, the significant absolute inequalities were seen in 2002 (10.0 [1.6–18.4]) and 2013 (9.6 [1.8–17.4]) (Table 4). We observed a similar pattern on the relative scale: moving from low-SES to high-SES was associated with a 1.5-fold increase in periodontal health's probability in 2002 and 2013. On the other hand, 1995 and 2007 did not show any significant inequality gap on absolute and relative inequality scales.

Compared with low-SES individuals, the prevalence of deep pockets in high-SES individuals was 4.2 percentage points lower in 1995 (Table 4). The relative scale finding was similar to the absolute inequality. The regression-based relative effect index decreased by almost 50% (0.216–0.869) in 1995, indicating that the high-SES individuals had half risk of deep pockets than those in the low-SES group. In contrast, we did not observe any significant absolute and relative inequality of deep pockets in 2002, 2007, and 2013.

### Trends in socioeconomic inequality of oral hygiene and periodontal status

**Fig. 2:**
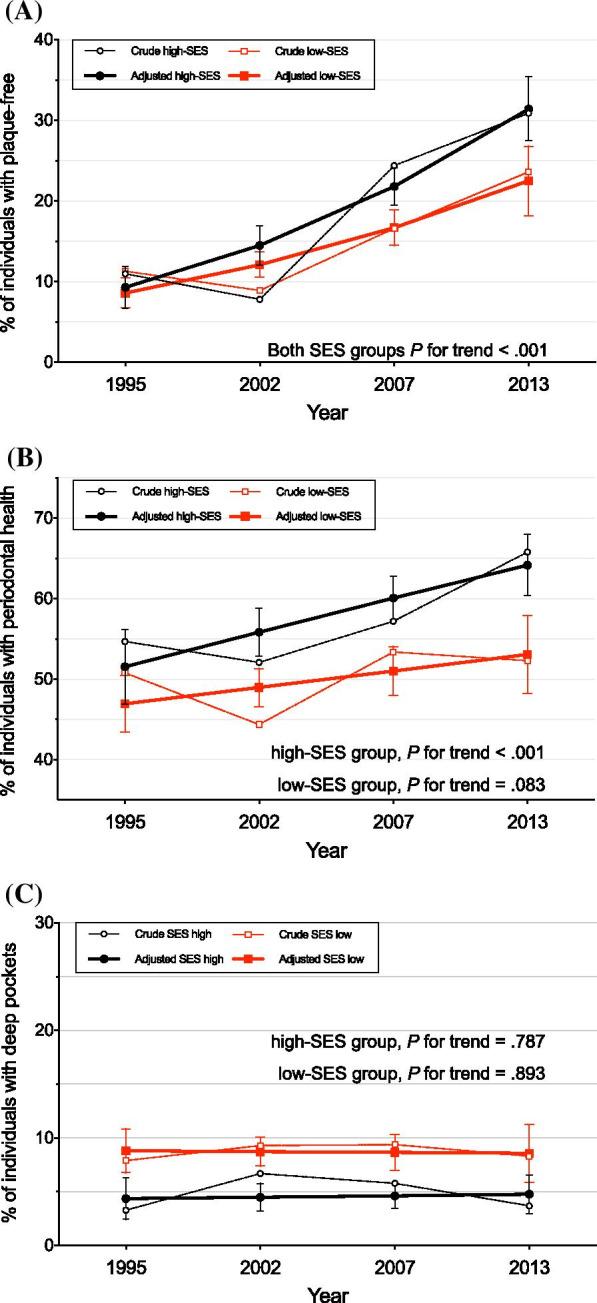
Adjusted percentage of plaque-free (**a**), periodontal health (**b**), and deep pockets (**c**) in adults aged 25–54 stratified by socioeconomic status from the Dutch National Oral Health Survey 1995–2013. All logistic regression models were adjusted for including age, gender, ethnicity, SES, time since the last dental visit, number of teeth present, and oral hygiene behaviors (toothbrushing frequency, floss usage, and toothpick usage). All *P* for trends were calculated using survey year as a continuous variable based on the multivariable regression model and with a two-sided significance level of .05

Figure 2a illustrates the trends over time in the crude and adjusted percentages of individuals without detected plaque (OHI-S = 0) by SES-groups. In the 1995–2013 period, there was a significant increase in the adjusted percentage of individuals with OHI-S = 0 in both the low- and high-SES groups (*P*_trend_ < 0.001, Table 5), after adjusting for potential confounders. The slope of the high-SES group's temporal trend was slightly steeper than the corresponding slope for the low-SES group (Fig. 2a). However, the interaction effect of survey year × SES on plaque-free did not reach a significant level (*P*_interaction_ = 0.198, Table 5).

**Table 5:** Trend analysis of plaque-free, periodontal health, and deep pockets in adults aged 25–54 from the Dutch National Oral Health Survey 1995–2013

Adjusted OR (95% CI)	Plaque-free	Periodontal health	Deep pockets
*Total population*			
Survey year			
1995	1 [reference]	1 [reference]	1 [reference]
2002	0.752 (0.539 to 1.048)	0.893 (0.733 to 1.090)	1.155 (0.792 to 1.686)
2007	**2.073 (1.546 to 2.779)**	**1.289 (1.043 to 1.593)**	1.102 (0.728 to 1.669)
2013	**3.158 (2.372 to 4.206)**	**1.542 (1.242 to 1.913)**	0.883 (0.562 to 1.388)
*P* for temporal trend ^a^	.000	.000	.652
*Low-SES population*			
Survey year			
1995	1 [reference]	1 [reference]	1 [reference]
2002	0.754 (0.506 to 1.123)	.817 (0.645 to 1.036)	1.071 (0.703 to 1.631)
2007	**1.730 (1.136 to 2.634)**	1.248 (0.935 to 1.665)	1.038 (0.626 to 1.722)
2013	**2.915 (1.914 to 4.438)**	1.259 (0.922 to 1.718)	0.882 (0.502 to 1.548)
*P* for temporal trend ^a^	.000	.083	.893
*High-SES population*			
Survey year			
1995	1 [reference]	1 [reference]	1 [reference]
2002	0.669 (0.353 to 1.266)	1.075 (0.741 to 1.559)	1.633 (0.690 to 3.865)
2007	**2.525 (1.644 to 3.879)**	1.359 (0.988 to 1.869)	1.410 (0.646 to 3.080)
2013	**3.501 (2.316 to 5.292)**	**1.793 (1.312 to 2.449)**	1.061 (0.470 to 2.397)
*P* for temporal trend ^a^	.000	.000	.787
*P* for interaction ^b^	.198	.490	.678

All logistic regression models were adjusted for including age, gender, ethnicity, SES, time since the last dental visit, number of teeth present, and oral hygiene behaviors (toothbrushing frequency, floss usage, and toothpick usage). Low-SES was regarded as reference

^a^All P for trends were calculated using survey year as a continuous variable

^b^The interaction term indicated survey year × SES

Bold indicates *P* value < .05

The secular trends in the crude and adjusted percentage of periodontal health (PDI = 0) are shown in Fig. 2b. After adjusting for confounders, the upward trends with respect to periodontal health were identified, with a percentage increase in the high-SES group (*P*_trend_ < 0.001, Table 5). In contrast, the percentage of periodontal health among the low-SES participants remained relatively constant (*P*_trend_ = 0.083, Table 5). Although SES inequality in periodontal health tends to widen increasingly over the last two decades (Fig. 2b), the SES group-by-time interaction did not reach statistical significance (*P*_interaction_ = 0.490, Table 5).

There was little difference in the adjusted percentage of individuals with deep pockets (PDI = 2) during all survey years, either in the high-SES or low-SES group (Fig. 2c). In the multivariable logistic regression models, no significant temporal trends were found for deep pockets between 1995 and 2013 (low-SES: *P*_trend_ = 0.893; high-SES group: *P*_trend_ = 0.787, Table 5). Additionally, the interaction term survey year × SES was not statistically significant (*P*_interaction_ = 0.678, Table 5).

## Discussion

This study tracked the trends in oral hygiene and periodontal status over the last two decades in a large sample of Dutch adults. Our data indicate that oral hygiene significantly improved over time in both the low- and high-SES groups and that the number of dental plaque-free individuals substantially increased as well. The improvement in periodontal health during the survey period sightly differed between high and low SES. In the low-SES group, the level of periodontal health remained almost constant. In contrast, our observations revealed a significant improvement in the percentage of periodontally healthy individuals in the high-SES population over time. Yet, within both SES groups, the prevalence of deep pockets over the last two decades remained the same.

We observed a significant decrease in the number of individuals with poor oral hygiene (OHI-S ≥ 2) since 1995. Concomitantly, there was an increase in the percentage of plaque-free individuals in the total sample. These findings regarding oral hygiene are similar to the trends identified in a previous Dutch dental health survey covering the period of 1983–1995 [18] and are aligned with the trends identified in studies conducted in other European countries. These findings reflect the remarkable improvements in oral hygiene that have occurred in most developed countries over the decades [4, 5, 7]. Notably, the improvements seen in oral hygiene among members of the low-SES group did not occur as rapidly as those in the high-SES group. These findings suggest that the previously noted persistence of socio-economic inequality was observed against a background of significantly improved oral hygiene over the last 20 years.

The current study focused on individuals with periodontal health, given that the main objective of dental care is to attain and maintain a healthy periodontium [36]. The definitions of periodontal disease that can be found among various clinical studies exhibit a high degree of heterogeneity [37]. Furthermore, the varying degrees of severity of periodontitis and differences in the range of teeth affected lead to a series of “gray zones” in the classification system [38]. In contrast, the population consisting of individuals who exhibit healthy seems to show more homogeneity. Thus, tracking the percentage of individuals who exhibit periodontal health could also be an acceptable alternative in periodontal epidemiology.

The novel contribution of this study is the insight that socioeconomic inequality in periodontal health tends to widen in the Netherlands over the last two decades. To minimize the impact of the potential confounding factors, we attempted to control for the covariates using multivariable regressions and afterward analyzed the differentials in SES. The low-SES population did not significantly improve in periodontal health over time compared to their high-SES counterparts (*P* for trend = 0.083). Periodontal status is subject to periodontally accumulative defects, complex comorbidities, and multiple host risk factors [39]. Thus, the efficiency of measures intended to maintain periodontal health inferior to that designed to control plaque, suggesting that periodontal health is more challenging to change the SES inequality.

In the current study, education was used as a proxy for SES, as it has been documented to impact the ability to acquire and interpret periodontal health-related information [40]. Additionally, the level of education is also linked to a person’s position in his or her respective social structure. Individuals occupying higher social positions are hypothesized to have higher levels of social support, more control at work, and more job security than those of lower backgrounds in terms of SES [41]. All of the differentials in the high-SES group identified above facilitate access to costly dental services, particularly in developed countries. Another possible explanation for the widening differentials in SES is the changes that have occurred in oral health preventive programs, especially the 2006 health insurance reform in the Netherlands. Despite the fact that the mandatory basic dental insurance provides universal access to limited dental treatments to all Dutch citizens, most dental care requires supplementary insurance or out-of-pocket payment [20]. We assume that these financial barriers are more likely to be present in the low-SES group and that members of this group will therefore have more unmet dental care needs, which may explain the lack of improvement in periodontal health over time [42].

We did not observe the percentages of individuals with pocket depth > 5.5 mm significantly decreased over time, even in the high-SES population. The prevalence of deep pockets in the current study was 8.7% (low-SES) and 4.6% (high-SES), with a similar age-standardized prevalence in the high-income countries (6.6%, CI [5.5–7.8%]) [2]. Moreover, the unchanged prevalence of deep pockets over decades has been reported in previous studies in the Netherlands [18] as well as the other countries [6, 7, 43]. In the US, the prevalence of deep pockets nearly remained the same from 13.1% (CI, 11.7–14.5%) in 1988 to 15.7% (CI, 13.8–17.5%) in 2012 [43]. Similarly, no consistent trend of decreasing prevalence of deep pockets during either three- or four-decade intervals was found in Sweden [6, 7].

Several limitations of the current study should be considered. Firstly, the Dutch National Oral Health Survey employs the PDI for defining periodontal health and disease. Pocket depth index could cause misclassification and biased estimation as attachment loss is not assessed and a partial mouth recording protocol was used [44, 45]. There has been an updated classification system and case definition incorporating gingival bleeding, pocket depth, and attachment loss [46–48]. We conducted the analyses based on two different categorizing periodontal status methods (PDI = 0 and PDI = 2) to overcome this limitation partially. Secondly, this study selected education level as a single proxy for SES despite it would have been an acceptable and valid indicator [49]. Beyond education, other indicators related to socioeconomic inequalities include job position, income, tangible resources, or a combination of these variables [41, 50]. Since this information was not included in the TNO database, these additional indicators of SES were not considered in the analyses. Thirdly, the generalizability of the results to older populations may be limited, given that only young and middle-aged adults were included in the study. As the prevalence of periodontal disease increases with age, our results cannot necessarily be generalized across all age groups. Without the elderly, the prevalence of deep pockets in the current study could be underestimated compared to overall Swedish or the US population [6, 7, 43].

## Conclusions

In conclusion, this study, using data from the Dutch National Oral Health Survey, demonstrated that socioeconomic inequalities in oral hygiene and periodontal status existed in the Dutch population in the last two decades. The trend in terms of improved periodontal health has not benefitted both SES groups equally, as the low-SES group has benefitted less than the high-SES group. The inequality identified in this study persisted between 1995 and 2013 in the Netherlands. It is suggested that oral health workers should be aware that low-SES populations should be provided with more preventive care and dental education. Oral and dental policymakers may need to increasingly target socioeconomic inequality and tailor appropriate policies for different SES groups.

## Data Availability

Dutch National Oral Health Survey series data have been deposited in the database developed by the Netherlands Organization for Applied Scientific Research (Toegepast Natuurwetenschappelijk Onderzoek, TNO [https://www.tno.nl/en/]). The data are not openly available due to subjects' confidentiality. The datasets used and/or analyzed during the current study will be made available upon reasonable request for academic use and within the limitations of the provided informed consent by the corresponding author upon acceptance.

## Abbreviations

SES: Socioeconomic status
OHI-S: Simplified Oral Hygiene Index
PDI: Pocket Depth Index
95% CI: 95% Confidence interval
TNO: Toegepast Natuurwetenschappelijk Onderzoek,
HIF: Health Insurance Fund
CPI: Community Periodontal Index
